# 3,5,6,7,8,3′,4′-Heptamethoxyflavone, a *Citrus* Flavonoid, Inhibits Collagenase Activity and Induces Type I Procollagen Synthesis in HDFn Cells

**DOI:** 10.3390/ijms19020620

**Published:** 2018-02-22

**Authors:** Hong-Il Kim, Yong-Un Jeong, Jong-Hyeon Kim, Young-Jin Park

**Affiliations:** Department of Biomedical Chemistry, Research Institute for Biomedical & Health Science, College of Biomedical and Health Science, Konkuk University, 268 Chungwon-daero, Chungju-si 27478, Korea; kwangdae7@kku.ac.kr (H.-I.K.); s11034@kku.ac.kr (Y.-U.J.); dhqksdl@kku.ac.kr (J.-H.K.)

**Keywords:** *Citrus unshiu*, collagenase, HDFn, Heptamethoxyflavone, Type I procollagen

## Abstract

*Citrus* fruits contain various types of flavonoids with powerful anti-aging and photoprotective effects on the skin, and have thus been attracting attention as potential, efficacious skincare agents. Here, we aimed to investigate the chemical composition of *Citrus unshiu* and its protective effects on photoaging. We isolated and identified a bioactive compound, 3,5,6,7,8,3′,4′-heptamethoxyflavone (HMF), from *C. unshiu* peels using ethanol extraction and hexane fractionation. HMF inhibited collagenase activity and increased type I procollagen content in UV-induced human dermal fibroblast neonatal (HDFn) cells. HMF also suppressed the expression of matrix metalloproteinases 1 (MMP-1) and induced the expression of type I procollagen protein in UV-induced HDFn cells. Additionally, HMF inhibited ultraviolet B (UVB)-induced phosphorylation of the mitogen-activated protein kinases (MAPK) cascade signaling components—ERK, JNK, and c-Jun—which are involved in the induction of MMP-1 expression. Furthermore, HMF affected the TGF-β/Smad signaling pathway, which is involved in the regulation of type I procollagen expression. In particular, HMF induced Smad3 protein expression and suppressed Smad7 protein expression in UV-induced HDFn cells in a dose-dependent manner. These findings suggest a role for *Citrus* *unshiu* in the preparation of skincare products in future.

## 1. Introduction

Skin aging, characterized by thickening, wrinkling, and roughness of the skin, is a complex process potentialing several aesthetic and functional changes. It can be divided into two main processes: intrinsic (programmed) aging and photoaging. Photoaging, or photodamage, caused by exposure to ultraviolet (UV) radiation from the sun (particularly UVA (315–400 nm) and UVB radiation (280–315 nm) [1,2]), results in premature skin aging. Chronic UV irradiation results in markedly increased reactive oxygen species (ROS) levels that trigger the release of proinflammatory cytokines and growth factors, and stimulate mitogen-activated protein kinases (MAPKs) such as extracellular signal-regulated kinase (ERK), p38 kinase, and c-Jun N-terminal kinase (JNK), which converge to stimulate activator protein-1 (AP-1) [3,4,5]. The activity of AP-1, a c-Jun and c-Fos heterodimer complex, is dependent on the degree of c-Fos expression and c-Jun phosphorylation [6,7]. Consequently, the transcriptional activation of AP-1 increases the production of matrix metalloproteinases (MMPs), leading to degradation of the collagen and elastin fibers of the extracellular matrix which provide structural support to the skin dermis [8]. There are 23 known MMPs identified in humans, and AP-1 binding sites are found in the promoter region of several inducible human MMP genes, including *MMP-1* (collagenase-1), *MMP-3* (stromelysin-1), *MMP-7* (matrilysin), *MMP-9* (gelatinase B), *MMP-10* (stromelysin-2), *MMP-12* (metalloelastase), and *MMP-13* (collagenase-3) [8,9]. In particular, increased AP-1 activity upregulates *MMP-1*, which is primarily responsible for degradation of the extracellular matrix (ECM) [6,9,10]. Transforming growth factor β (TGF-β), a multifunctional cytokine, induces fibroblast proliferation and collagen synthesis in the dermis [11,12,13,14]. Smad7 antagonizes TGF-β signaling by interacting with the TGF-β type I receptor (TβRI) and inhibiting activation of Smad2 and Smad3 [15,16]. 

Phenolics—secondary metabolites of plants—are naturally occurring compounds containing a phenol group comprising an aromatic ring with a hydroxyl substituent. They are found in herbs, fruits, vegetables, grain, tea, coffee beans, propolis, etc. [17]. Among the three important groups of phenolics for humans, flavonoids are benzo-γ-pyrone derivatives and low-molecular-weight polyphenolic substances. The other phenolics are phenolic acids and high-molecular-weight polyphenols [18,19]. Flavonoids are capable of scavenging reactive oxygen species (ROS) such as hydroxyl radicals, singlet oxygen, and superoxide anions [20,21,22]. Moreover, flavonoids inhibit cyclooxygenase (COX, EC 1.14.99.1), lipoxygenase (LOX, EC 1.13.11.-), mitochondrial succinate dehydrogenase (SDH, EC 1.3.3.99), monooxygenase (EC 1.14.13.-), NADH-oxidase (EC 1.6.3.1), phospholipase A2 (PLA, EC 3.1.1.4), protein kinases (EC 2.7.11.-), xanthine oxidase (XO, EC 1.17.3.2), and nuclear transcription factor (NF-κB) activities [23,24]. In addition, there have been several reports demonstrating the antiphotoaging activities of *Citrus* flavonoids, such as apigenin (4′,5,7-trihydroxyflavone), luteolin (3′,4′,5,7-tetrahydroxyflavone), nobiletin (5,6,7,8,3′,4′-hexamethoxyflavone), quercetin (3,3′,4′,5,7-pentahydroxyflavon), and wogonin (5,7-dihydroxy-8-methoxyflavone), which inhibit the UV-induced MAPK signaling pathway during photoaging [17,25,26,27,28]. Apigenin and luteolin inhibit MMP-1 expression in UVA-induced human keratinocytes [28]. Nobiletin inhibits photoaging in UVB-induced human keratinocytes [29]. Quercetin inhibits MMP-1 expression through the suppression of the MAPK signaling pathway in UVA-induced human dermal fibroblasts [18,19]. Wogonin inhibits MMP-1 expression in 12-*O*-tetradecanoylphobol 13-acetate (TPA)-induced human dermal fibroblasts [27].

As described above, flavonoids are powerful agents with antioxidant and anti-skin-aging effects, especially against photoaging. However, some flavonoids have also been found to be mutagenic in vitro through pro-oxidant, rather than antioxidant, actions of these compounds. Therefore, it is necessary to investigate their toxic effects before use in humans [30]. In this study, we isolated and identified 3,5,6,7,8,3′,4′-heptamethoxyflavone (HMF, methylated flavonoid) from the *Citrus unshiu* peel. HMF inhibited MMP-1 expression and collagen degradation through suppression of the MAPK signaling pathway in UVB-induced human dermal fibroblasts (HDFn) at noncytotoxic concentrations. These findings suggest that HMF may be beneficial in preventing UVB-induced oxygen free radical generation and is a useful agent for preventing skin damage and photoaging.

## 2. Results and Discussion

### 2.1. Chemical Structure and Cytotoxicity of HMF on HDFn Cells

**Figure 1:**
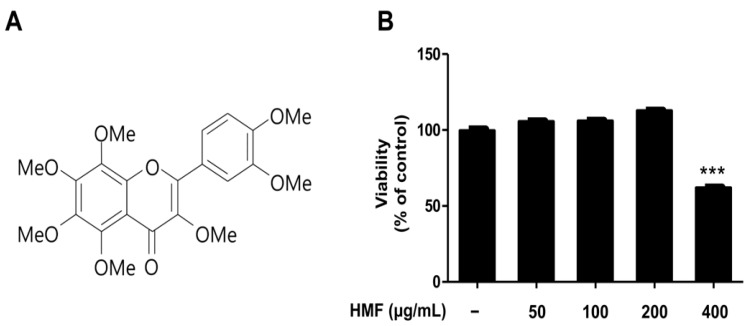
Chemical structure and cytotoxicity of 3,5,6,7,8,3′,4′-heptamethoxyflavone (HMF) isolated from *C*. *unshiu* peel. (**A**) Chemical structure and (**B**) cytotoxic effects of HMF on human dermal fibroblast neonatal (HDFn) cells. HDFn cells were treated with HMF (50, 100, 200, and 400 μg/mL) for 24 h. Values are means ± standard deviation (SD) of three independent experiments and relative to percentages of control cells. Statistical significance of differences was evaluated using one-way analysis of variance (ANOVA) followed by Tukey’s test. *** *p* < 0.001 versus HDFn cells without HMF treatment.

Flavonoids are major bioactive compounds in *Citrus* fruits. There are six classes of flavonoids: anthocyanins, flavanols (or catechins), flavanones, flavones, flavonols, and isoflavones [31]. Initially, a 50% ethanol extract of dried *C. unshiu* peel was suspended in water and partitioned successively with hexane. The bioactive compound was identified as HMF by comparing its spectroscopic nuclear magnetic resonance (NMR) data with those previously reported in the literature (Figure 1A, Figure S1 and S2, see also Materials and Methods Section 3.2) [32,33,34]. 

Although they have been widely reported to have beneficial effects on health, several studies suggest that flavonoids also have mutagenic effects due to their pro-oxidant activities [30,35,36]. Moreover, high intakes of flavonoids may affect the activity of key enzymes in hormone metabolism due to their diverse properties [30]. For example, myricetin, quercetagetin, and quercetin caused respiratory bursts in mitochondria and underwent autoxidation resulting in ROS (hydrogen peroxide, hydroxyl radical, and superoxide) formation [37]. It has also been reported that daidzein, genistein, kaempferol, naringenin, and quercetin inhibit thyroxine synthesis and thyroid peroxidase (TPO), which play important roles in thyroid hormone synthesis [38]. In addition, Matsuo et al. [39] indicated that flavonoids can exert toxic effects on human lung embryonic fibroblasts (TIG-1) and human umbilical vein endothelial cells (HUVECs) at relatively high concentrations, even though they showed beneficial effects at relatively low concentrations. For this reason, we examined the cytotoxic effects of HMF on HDFn cells treated with the indicated concentrations (50, 100, 200, and 400 μg/mL) for 24 h. As shown in Figure 1B, HMF showed no cytotoxic effects on HDFn cells up to 200 μg/mL. However, cell viability was significantly affected by HMF at 400 μg/mL. This concentration resulted in approximately 50% HDFn cell death by when compared with untreated cells. Therefore, HDFn cells were treated with HMF at concentrations of 50, 100, and 200 μg/mL in subsequent experiments. 

### 2.2. Effects of HMF on Collagenase Activity and Type I Procollagen Contents of HDFn Cells

Type I collagen, the most abundant fibrillar type collagen, is the main structural component of the extracellular matrix (ECM). Type I procollagen, a soluble precursor of type I collagen, is synthesized and secreted from fibroblasts then proteolytically cleaved by procollagen N- and C-proteases to form collagen fibrils [40]. Matrix metalloproteases (MMPs)—homogeneous zinc-dependent endopeptidases—can degrade all components of the ECM [9]. Among the MMP family, including gelatinases, matrilysins, membrane-type MMPs, stromelysins, and nonclassified MMPs, collagenases are capable of degrading native collagen fibrils in the ECM. Thus, collagenase is a key enzyme in the degradation of collagen in normal connective tissue [41]. For this reason, natural compounds that inhibit collagenase activity and consequently prevent collagen degradation may be potential candidates for preventing skin photoaging and photodamage. Flavonoids are known to have powerful anti-aging and photoprotective effects on the skin. Over 60 types of flavonoids including anthocyanins, flavanones, flavans, flavones, and flavonols have been identified from *Citrus* fruits [31]. A direct inhibitory effect of flavonoids on collagenase has rarely been demonstrated, despite the importance of collagenase and collagen breakdown in photoaging and photodamage. It has only been described that epicatechin gallate (ECG) and epigallocatechin gallate (EGCG), isolated from tea and *Citrus* flavonoids including hesperidin, hesperetin, and naringenin, inhibit collagenase [42,43]. Moreover, *Citrus* peel and seeds are very rich in flavonoids, particularly the peel [44]. We therefore investigated the inhibitory activity of HMF, isolated from *C. unshiu* peel, on cellular collagenase activity in HDFn cells. 

**Figure 2:**
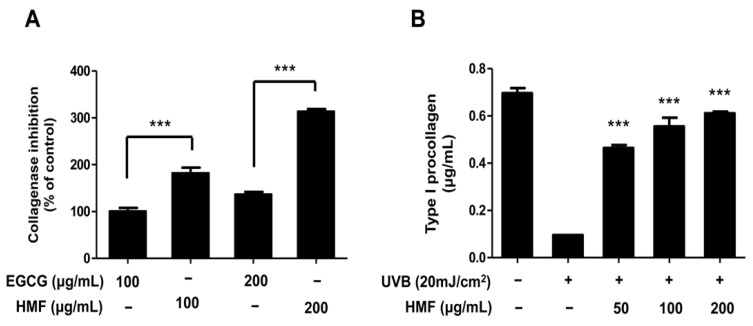
Effects of 3,5,6,7,8,3′,4′-heptamethoxyflavone (HMF) on cellular collagenase activity and type I procollagen content of HDFn cells. (**A**) Cellular collagenase inhibitory activity and (**B**) type I procollagen content in UVB-induced HDFn cells. Values are means ± standard deviation (SD) of three independent experiments. Statistical significance of differences was evaluated using one-way analysis of variance (ANOVA) followed by Tukey’s test. *** *p* < 0.001 versus HDFn cells without HMF treatment.

Comparing the collagenase inhibitory activities of EGCG and HMF, treatment with HMF showed inhibitory activity approximately two times higher than that of EGCG at the same concentrations (Figure 2A). In addition, we examined the effects of HMF on collagen degradation, which was shown to decrease in UVB-induced HDFn cells after treatment with HMF (Figure 2B). Treatment with HMF significantly increased the type I procollagen content of HDFn cells in a dose-dependent manner. The amount of the type I procollagen in UVB-irradiated HDFn cells was increased to 0.46 ± 0.01, 0.56 ± 0.04, and 0.61 ± 0.01 μg/mL by pretreatment with HMF at 50, 100, and 200 μg/mL concentrations, respectively (Figure 2B). These findings indicate that HMF significantly inhibited collagenase activity and consequently prevented collagen degradation in UVB-induced HDFn cells by 87% (at 200 μg/mL of HMF) suggesting that HMF may protect against skin photoaging and photodamage (Figure 2B).

### 2.3. Effects of HMF on Cellular MMP-1 and Type I Procollagen Expression in HDFn Cells

Among the MMPs, MMP-1 (collagenase-1) was the first identified enzyme primarily responsible for degradation of the extracellular matrix (ECM) in UV-irradiated dermal tissues [6,9,10]. Active compounds possessing the ability to inhibit the collagen degradation enzymes (such as MMP-1) and promote collagen synthesis have possible uses in cosmetic and therapeutic agents to prevent photoaging and photodamage. It was previously reported that several bioactive compounds such as apigenin, luteolin, nobiletin, quercetin, and wogonin from *Citrus* inhibit MMP-1 expression in UV-induced human keratinocytes or human dermal fibroblasts [17,25,26,27,28]. Therefore, in the present study, the effects of HMF on MMP-1 and type I procollagen expression in UV-induced HDFn cells were also examined.

**Figure 3:**
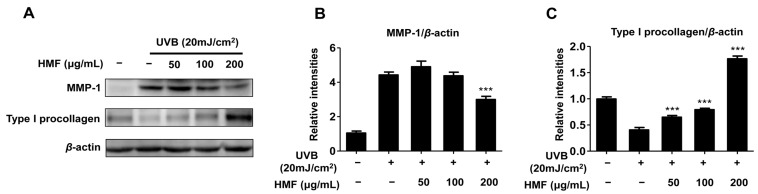
Effects of 3,5,6,7,8,3′,4′-heptamethoxyflavone (HMF) on cellular matrix metalloproteinases 1 (MMP-1) protein and type I procollagen protein expression in HDFn cells. HDFn cells were pretreated with HMF (50, 100, and 200 μg/mL) for 24 h following treatment with UVB irradiation (20 mJ/cm^2^). (**A**) Cellular protein levels were examined using western blot analysis; (**B**) Relative protein expression levels of MMP-1 protein and (**C**) type I procollagen protein. Values are means ± standard deviation (SD) of three independent experiments. Statistical significance of differences was evaluated using one-way analysis of variance (ANOVA) followed by Tukey’s test. *** *p* < 0.001 versus HDFn cells without HMF treatment.

Western blot analysis showed that HMF inhibited the expression of MMP-1 in UV-induced HDFn cells (Figure 3). The induction of this protein by UVB (20 mJ/cm^2^) was significantly suppressed by pretreatment with HMF at a concentration of 200 μg/mL (Figure 3B). Our result also showed that the expression of type I procollagen was markedly increased by pretreatment with HMF in the UV-induced HDFn cells in a dose-dependent manner (Figure 3C). The relative expression of type I procollagen after pretreatment with 200 μg/mL of HMF was about 5.5 times higher than that of nontreated UV-induced HDFn cells (Figure 3C). These findings indicate that HMF significantly downregulates MMP-1 expression, whereas it upregulates type I procollagen expression in UV-induced HDFn.

### 2.4. Effects of HMF on MAPK Signaling Pathways in HDFn Cells

Degradation of the extracellular matrix (ECM) by UV irradiation has been well studied in human skin tissue [3,4,5]. UV irradiation produces reactive oxygen species (ROS), which converge to stimulate the activator protein-1 (AP-1) through mitogen-activated protein kinases (MAPKs) such as extracellular signal-regulated kinase (ERK), p38 kinase, and c-Jun N-terminal kinase (JNK) [3,4,5]. In addition, the degree of c-Fos expression and c-Jun phosphorylation affect the activity of AP-1, which triggers the production of matrix metalloproteinases (MMPs) [6,7]. It was also previously reported that *Citrus* flavonoids such as apigenin, luteolin, nobiletin, and quercetin suppress the phosphorylation of the MAPK pathway components in UV-induced human keratinocytes or human dermal fibroblasts [17,25,26,27,28]. Activation of ERK and JNK have been reported to phosphorylate c-Jun, suggesting cross-talk between ERK and JNK in the regulation of c-Jun activity [6,45,46]. It has been reported that p38 contributes to AP-1 activity by activating transcription factors such as transcription factor 2 (ATF2), Ets-like transcription factor 1 (Elk1), and SRF accessory protein 1 (SAP1), which upregulate c-Jun and c-Fos promoter activity [47]. In addition, AP-1 activity is dependent on the abundance of c-Jun and c-Fos as well as their degree of phosphorylation [6,7]. 

**Figure 4:**
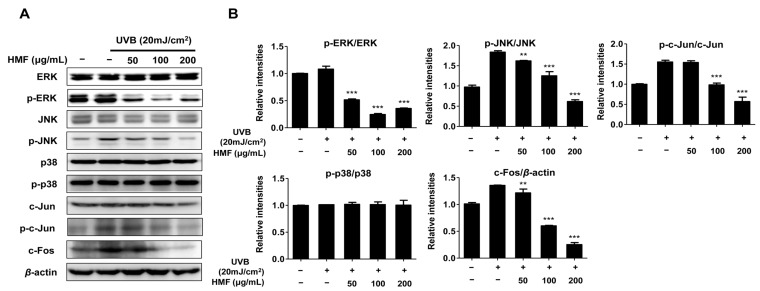
Effects of 3,5,6,7,8,3′,4′-heptamethoxyflavone (HMF) on mitogen-activated protein kinase (MAPK) signaling components in HDFn cells. HDFn cells were treated with HFM (50, 100, and 200 μg/mL) for 24 h following treatment with UVB irradiation (20 mJ/cm^2^). Cellular protein levels were examined using (**A**) western blot analysis and (**B**) relative expression levels were calculated using the TL-100 software program (TotalLab, Newcastle, UK). Values are means ± standard deviation (SD) of three independent experiments. Statistical significance of differences was evaluated using one-way analysis of variance (ANOVA) followed by Tukey’s test. ** *p* < 0.01 and *** *p* < 0.001 versus HDFn cells without HMF treatment.

In this study, we evaluated the phosphorylation levels of MAP kinases including ERK, p38, JNK, and c-Jun in UV-induced HDFn cells pretreated with HMF. As shown in Figure 4, the phosphorylation of ERK and JNK proteins were significantly decreased by treatment with HMF at all concentrations. The phosphorylation of c-Jun protein was also significantly decreased by treatment with HMF at concentrations of 100 and 200 μg/mL. Our results also show that neither phosphorylation nor expression of the p38 protein is affected by HMF at all concentrations (Figure 4). However, the expression of c-Fos was significantly inhibited by treatment with HMF at concentrations of 50, 100, and 200 μg/mL. These results indicate that the inhibition of MMP-1 expression by HMF is associated with MAPK family proteins including ERK, JNK, and c-Jun. In particular, HMF induced the phosphorylation of ERK, JNK, and c-Jun. Although the phosphorylation of the p38 protein was not inhibited, HMF suppressed the expression of the c-Fos protein.

### 2.5. Effects of HMF on Smad3 and Smad7 Expression in HDFn Cells

**Figure 5:**
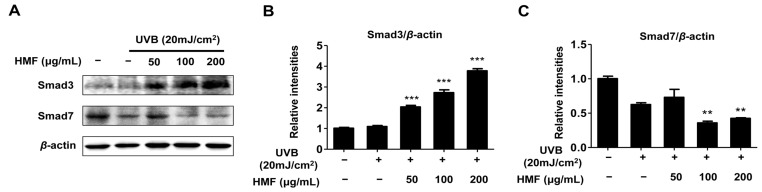
Effects of 3,5,6,7,8,3′,4′-heptamethoxyflavone (HMF) on expression of Smad3 and Smad7 protein in HDFn cells. HDFn cells were treated with HFM (50, 100, and 200 μg/mL) for 24 h following treatment with UVB irradiation (20 mJ/cm^2^). Cellular protein levels were examined using (**A**) western blot analysis and (**B**) relative expression levels were calculated using the TL-100 software program (TotalLab, Newcastle, UK). Values are means ± standard deviation (SD) of three independent experiments. Statistical significance of differences was evaluated using one-way analysis of variance (ANOVA) followed by Tukey’s test. ** *p* < 0.01 and *** *p* < 0.001 versus HDFn cells without HMF treatment.

UV irradiation influences not only the degradation of the extracellular matrix (ECM) through MAP kinase signaling but also the inhibition of collagen synthesis by the TGF-β/Smad pathway [3,4,5,40]. Once TGF-β binds to the cell surface receptor (TβRI and TβII), the TGF-β receptor complex initiates its cellular action by transiently interacting with the receptor-regulated Smad proteins resulting in their phosphorylation. Phosphorylated Smad2/Smad3 combine with Smad4 and translocate into the nucleus to regulate transcription of TGF-β-regulated genes including type I procollagen [48]. Decreased expression of the type I procollagen gene was observed in skin fibroblasts, which interfered with TGF-β, Smad3, or Smad4 expression [49,50,51,52,53]. Smad7 interacts with TβRI to prevent phosphorylation and activation of Smad2 and Smad3 which interrupt TGF-β signaling [15,16]. Therefore, we hypothesized that HMF-induced type I procollagen upregulation is regulated by the TGF-β/Smad signal transduction cascade. As expected, the expression of Smad3 protein was dose-dependently increased in the UV-induced HDFn cells by pretreatment with HMF (Figure 5). In addition, Smad7 protein expression was significantly decreased by pretreatment with HMF at concentrations of 100 and 200 μg/mL (Figure 5C). Although the TGF-β expression level was not determined in the present study, these results suggest that HMF contributed to the induction of type I procollagen expression through the TGF-β/Smad pathway.

## 3. Materials and Methods

### 3.1. Reagents

Dulbecco’s modified Eagle’s medium (DMEM), fetal bovine serum (FBS), antibiotics (penicillin and streptomycin), and other cell culture reagents were obtained from Gibco BRL (Grand Island, NY, USA). Primary antibodies against ERK, p-ERK, p38, p-p38, JNK, p-JNK, c-Jun, p-c-Jun, c-Fos, Smad3, Smad7, type I procollagen, MMP-1, actin, and horseradish peroxidase (HRP)-conjugated goat anti-mouse IgG secondary antibodies were purchased from Santa Cruz Biotechnology (Dallas, TX, USA). 3-(4,5-Dimethylthiazol-2-yl)-2,5-diphenyltetrazolium bromide (MTT) and all other chemicals were purchased from Sigma-Aldrich (St. Louis, MO, USA).

### 3.2. Isolation of 3,5,6,7,8,3′,4′-Heptamethoxyflavone

Dried *C. unshiu* peel (20 g) was crushed and extracted with 50% EtOH (5 L) at room temperature for 7 days. After evaporating the solvent in vacuum, the extract (2 g) was mixed with water (1 L) and extracted four times with hexane (1 L) to obtain the hexane-soluble fraction (840 mg). This fraction was separated on a silica gel (230–400 mesh, Merck Millipore, Darmstadt, Germany) column using n-hexane/EtOAc (gradient, 4:1, 1.5:1, 1:1.5, 1:2, and 1:4), and then analyzed. The 160 n-hexane/EtOAc fractions were separated using thin layer chromatography (TLC) to obtain 9 fractions (Fr. 1–9). Using bioassay-guided fractionation, Fr. 6 (43.5 mg) was subsequently purified using reverse phase (RP) C-18 (Merck Millipore, Darmstadt, Germany) column chromatography with methanol/water (6:1, 9:1, 13:1, and 19:1) at a flow rate of 1.5 mL/min and analyzed on TLC to obtain HMF (14.6 mg, purity > 98.0%). The HMF was finally identified by comparing the spectroscopic NMR data with a previously isolated sample [32,33,34]. HMF was dissolved in dimethyl sulfoxide (DMSO) for experimental use. 

HMF showed the following characteristics: Colorless, C_22_H_24_O_9_.

Proton (^1^H)-NMR (CDCl_3_, 400 MHz): δ 3.79, 3.81, 3.84, 3.85, 3.86, 3.95, 4.02 (each 3H, s, OCH_3_), 7.19 (^1^H, d, *J* = 11.6 Hz, H-5′), 7.65 (^1^H, d, *J* = 2.4 Hz, H-2′), and 7.70 (^1^H, dd, *J* = 2.4 Hz, 11.2 Hz, H-6′). ^13^C-NMR (75 MHz, CDCl3): δ 150.9 (C_2_), 140.0 (C_3_), 172.3 (C_4_), 143.3 (C_5_), 137.4 (C_6_), 150.9 (C_7_), 137.4 (C_8_), 147.3 (C_9_), 114.5 (C_10_), 122.5 (C-_1′_), 110.7 (C_2′_), 148.5 (C_3′_), 152.4 (C_4′_), 111.7 (C_5′_), 121.5 (C_6′_), 61.9 (3-OCH_3_), 61.8 (5-OCH_3_), 61.6 (6-OCH_3_), 61.4 (7-OCH_3_), 59.3 (8-OCH_3_), 55.6 (3′-OCH_3_), and 55.4 (4′-OCH_3_) [30].

### 3.3. Cell Culture

Primary human dermal fibroblast neonatal (HDFn) cells (PCS-201-010, American Type Culture Collection (ATCC), Manassas, VA, USA) were maintained and grown in DMEM containing 10% FBS and 1% antibiotics (penicillin and streptomycin) (all from Thermo Fisher Scientific Inc., Waltham, MA, USA). The cells were incubated in a humidified (95% air and 5% CO_2_) incubator at 37 °C. 

### 3.4. Cell Viability Assay

HDFn cell viability was analyzed using an MTT assay [54]. Cells were incubated (90% confluency) with various concentrations of the HMF (50, 100, 200, and 400 μg/mL) for 24 h. To determine cell viability, 0.5 mg/mL of MTT solution was added to each well and incubated with a cell suspension for 3 h. The supernatants were discarded and the resulting formazan crystals were dissolved in dimethyl sulfoxide (DMSO). Absorbance (570 nm) was measured using a microplate reader (TECAN, Männedorf, Switzerland).

### 3.5. Collagenase Activity Assay

Collagenase activity was measured using a slightly modified method based on Ha et al. [55]. HDFn cells were cultured in DMEM containing 10% FBS and incubated at 37 °C in a humidified 5% CO_2_ incubator. After incubation, the cells (~90% confluency) were harvested and washed using phosphate-buffered saline (PBS, pH 7.4). The cell pellets were suspended in 0.1 M Tris-HCl (pH 7.4) containing 0.1% Triton X-100 and 1 mM phenylmethylsulfonyl fluoride (PMSF), then disrupted by sonication for 5 min. Each tube contained HMF (100 and 200 μg/mL), 125 μL of 0.1 M Tris-HCl (pH 7.5) including 4 mM CaCl_2_, 4-phenylazo-benzyloxy-carbonyl-Pro-Leu-Gly-Pro-D-Arg (Pz-peptide), and 75 μL of the cell-free supernatant. The tube was incubated at 37 °C for 20 min, and the reaction was stopped by addition of 250 μL stop solution (6% citric acid). The reaction tube was mixed with 750 μL ethyl acetate and the absorbance of the supernatant (200 μL) was measured at 320 nm using a microplate reader. Collagenase activity was measured according to the following formula: collagenase inhibitory activity (%) = {1 − (OD_320_ of sample/OD_320_ of control)} × 100.

### 3.6. Type I Procollagen Assay

Type I procollagen content was determined by Procollagen Type I C-Peptide EIA Kit (Takara, Seoul, Korea). HDFn cells were seeded in 6-well plates at 7 × 10^5^ cells/well and allowed to attach for 24 h. HDFn cells were incubated with various concentrations of the HMF (50, 100, and 200 μg/mL) for 24 h in FBS-free media. Cells washed with phosphate-buffered saline (PBS, pH 7.4) were irradiated with UVB (20 mJ/cm^2^, Vilber Lourmat, Marne La Vallée, France) and then further incubated for 24 h at 37 °C in a humidified 5% CO_2_ incubator. After centrifugation at 10,000× *g* for 5 min, the 1/5 diluted cell-free supernatant was mixed with 100 μL antibody–horseradish peroxidase (POD) conjugate solution and then incubated for 3 h at 37 °C in an antibody-coated 96-well microtiter plate (Procollagen Type I C-Peptide EIA Kit, Takara). The cells were rinsed four times with phosphate-buffered saline (PBS, pH 7.4) and incubated for 15 min in the dark in substrate solution (H_2_O_2_ and tetramethylbenzidine) (Procollagen Type I C-Peptide EIA Kit, Takara). The reaction was stopped with 100 μL H_2_SO_4_ (1 N) and absorbance was measured at 450 nm. A standard was prepared using the serum concentration of procollagen type I C-peptide and the type I procollagen content was expressed as μg/mL.

### 3.7. Western Blot Analysis

Western blot was performed by using a previously described method [56]. Cell pellets were resuspended in lysis buffer (radioimmunoprecipitation assay buffer, Thermo Scientific, Seoul, Korea) containing 50 mM Tris (pH 7.4), 150 mM sodium chloride, 1 mM ethylenediaminetetraacetic acid, and 1% NP40. The total protein concentration was determined with the Bradford assay reagent (Bio-Rad, Philadelphia, PA, USA). Equal amounts of protein were electrophoresed using sodium dodecyl sulfate–polyacrylamide gel electrophoresis (SDS-PAGE) and transferred to a Hybond-enhanced chemiluminescence nitrocellulose membrane (Bio-Rad). The membrane was saturated with 3% bovine serum albumin (BSA) and incubated with 1:1000 diluted primary antibody (ERK, p-ERK, p38, p-p38, JNK, p-JNK, c-Jun, p-c-Jun, c-Fos, Smad3, Smad7, type I procollagen, and MMP-1) at 4 °C, overnight. Western signals were visualized using HRP-conjugated secondary antibodies and developed with enhanced chemiluminescence, and then quantified using the TL-100 software program (TotalLab, Newcastle, UK). 

### 3.8. Statistical Analysis

All data are presented as mean ± standard deviation (SD) of three independent experiments. Statistical significance between groups was evaluated using a one-way analysis of variance (ANOVA) followed by Tukey’s test using Prism (GraphPad Software Inc., La Jolla, CA, USA). 

## 4. Conclusions

The results of the present study suggest that HMF, a flavonoid compound isolated from *C. unshiu* peel, effectively protected against UV-induced photoaging of HDFn cells. We found that HMF inhibited collagenase activity and increased the type I procollagen content in UV-induced HDFn cells. It also suppressed MMP-1 and induced type I procollagen expression. Moreover, HMF affected the MAPK signaling pathway, which may contribute to the reduction in MMP-1 expression. UV irradiation influenced not only the degradation of the extracellular matrix (ECM) through MAP kinase signaling but also the inhibition of collagen synthesis through the TGF-β/Smad pathway. In the UV-induced HDFn cells, HMF affected the TGF-β/Smad signaling pathway. HMF induced Smad3 expression and suppressed Smad7 expression. 

This is the first report to show that HMF has a photoprotective effect in UV-induced HDFn cells through the phosphorylation of MAPK signals, such as ERK, JNK, and c-Jun, followed by the suppression of MMP-1 expression. Although further studies are required to elucidate an anti-photoaging effect of HMF in HDFn cells irradiated with different light sources including solar and UVA, this bioactive compound warrants further investigation for development as a potential agent in cosmetic products. 

## Supplementary Materials

The following are available online at http://www.mdpi.com/1422-0067/19/2/620/s1.
