# Supplementary figures and images for: 3,5,6,7,8,3′,4′-Heptamethoxyflavone, a Citrus Flavonoid, Inhibits Collagenase Activity and Induces Type I Procollagen Synthesis in HDFn Cells
Source: Int J Mol Sci. 2018 Feb 22;19(2):620. doi: 10.3390/ijms19020620 (PMC5855842; doi:10.3390/ijms19020620)

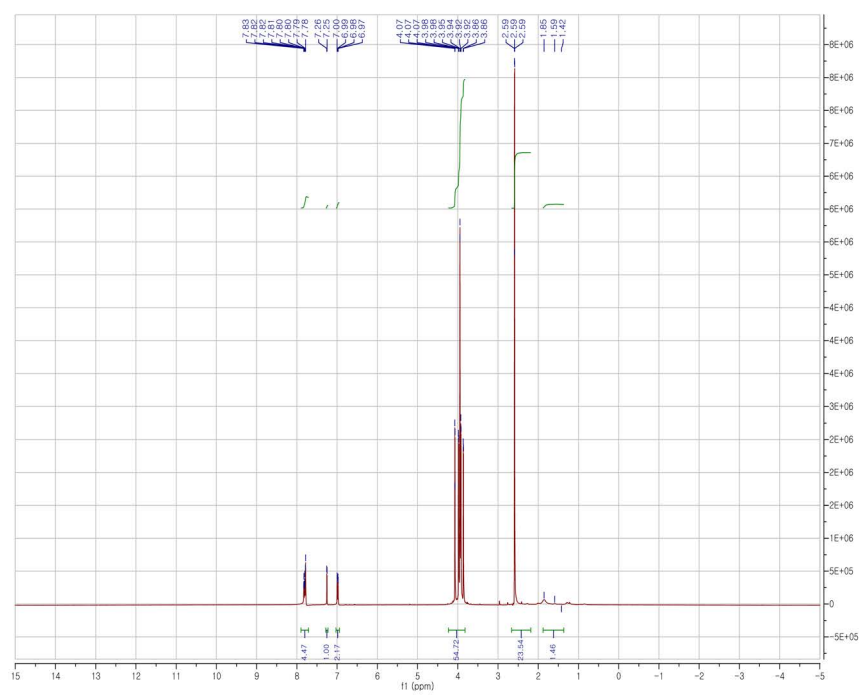

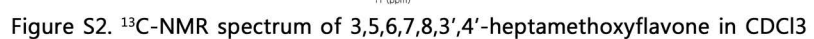

Figure S2.  $^{13}\text{C}$ -NMR spectrum of 3,5,6,7,8,3',4'-heptamethoxyflavone in  $\text{CDCl}_3$

Supplement: Supplementary file 1 [file ijms-19-00620-s001.pdf]
